# Exercise is associated with lower all-cause mortality among older adults: a national cohort survival analysis with county-level industrial air pollution indicators

**DOI:** 10.3389/fpubh.2026.1894835

**Published:** 2026-09-04

**Authors:** Wenxing Wang, Jiaqi Xu, Chenxi Wang, Yuanhui Zhao, Hong Ren

**Affiliations:** 1 School of Sport Science, Beijing Sport University, Beijing, China; 2 Key Laboratory of Exercise and Physical Fitness, Ministry of Education, Beijing Sport University, Beijing, China; 3 School of Physical Education, Xizang Minzu University, Xianyang, China

**Keywords:** all-cause mortality, cox proportional hazards model, exercise, industrial air pollution, older adults

## Abstract

**Background:**

Accelerated global population aging has increased the urgency of identifying effective non-pharmacological strategies to reduce mortality risk among older adults. Although exercise serves as a key protective factor, industrial air pollution may attenuate its health benefits, and this interaction remains unclear. This study aims to investigate whether county-level industrial air pollution indicators modify the association between exercise and all-cause mortality among older adults.

**Methods:**

A longitudinal survival analysis was performed using the Chinese Longitudinal Healthy Longevity Survey (2008–2014) with multistage stratified sampling across 23 provinces. The analysis included 16,954 participants aged ≥ 65 years with three follow-up waves spanning 6 years. Self-reported current exercise habits, survival outcomes, health status, sex, smoking status, baseline age, residence, body mass index, functional status, and comorbidity count were extracted. County-level industrial air pollution indicators (annual soot and SO_2_ emissions, and removals) were matched to individuals according to their residence. All pollution indicators were standardized, and time-varying covariates were updated at each wave. Cox proportional hazards models were constructed sequentially: univariate models for core variables (exercise habits and industrial air pollution indicators), two-way interaction models, and multivariate models with sequential adjustment for covariates (sex, health status, baseline age, smoking status, residence, BMI, functional status, and comorbidity count).

**Results:**

Self-reported current exercise habits were significantly associated with lower all-cause mortality risk after multivariate adjustment (fully adjusted HR = 0.835, 95% CI: 0.773–0.902, *p* < 0.001). SO_2_ emissions showed a weak association with survival risk in univariate analysis (HR = 1.023, 95% CI: 0.942–1.111, *p* = 0.585). The association estimate was further attenuated after multivariate adjustment (fully adjusted HR = 1.001, 95% CI: 0.874–1.146, *p* = 0.992). Soot-related indicators showed no significant mortality associations. The interaction between exercise habits and each of the four industrial air pollution indicators was not significant (all *p* > 0.05).

**Conclusion:**

Self-reported current exercise habits were consistently associated with lower all-cause mortality among Chinese older adults, and this association was robust to adjustment for covariates. No statistically significant multiplicative interaction was detected between exercise habits and any of the four pollution indicators. Continued attention to industrial emission control remains warranted, while promoting habitual exercise among older adults may be beneficial in areas with industrial emissions.

## 1 Introduction

Accelerated global population aging has made the health of older adults a major public health challenge. Exercise serves as a crucial non-pharmacological intervention to reduce mortality risk among older adults. However, exposure to industrial air pollution may reduce the health benefits that older adults gain from exercise. Current evidence indicates that regular moderate-to-vigorous physical activity (MVPA) significantly reduces all-cause mortality and cardiovascular disease risk in older adults (1–8). As little as 10–15 min of MVPA per week has been associated with health benefits, and increasing the activity volume to 250 min per week further enhances the protective effect of physical activity (PA) (1). For older adults with multiple chronic conditions, maintaining a high level of PA can reduce premature death risk by threefold (7). Moreover, older adults who follow the 24-h PA guidelines show a lower mortality rate (6). Collectively, these findings underscore the vital role of exercise in geriatric health management. However, widespread industrial air pollution may weaken exercise-induced health benefits (9). Chronic exposure to air pollutants such as PM_10_ and NO_2_ exhibits a significant dose–response relationship with increased cardiopulmonary mortality (10). Notably, the positive correlation between SO_2_ exposure and hypertension incidence appears more pronounced among physically active individuals (11). Individuals with higher exercise frequencies may experience greater pollution-related harm due to increased respiratory exposure (8, 12). Additionally, air pollutants may increase the risk of diseases such as metabolic syndrome by exacerbating systemic inflammatory responses (13). These observations suggest a potential interaction between exercise and air pollution exposure.

The interactive effects of exercise and industrial air pollution exposure on all-cause mortality among older adults remain unclear, with limited evidence from large-scale survival analyses. Although some epidemiological studies have found that a high PA level may partially offset the harmful effects of air pollution on blood pressure (14, 15), the relationship between these factors remains contentious (16, 17). Moreover, most existing studies used baseline PA level as a fixed covariate. However, given the uncertain nature of exercise behavior, using a fixed baseline measure of PA in a Cox proportional hazards model for a cohort study with long follow-up may be inappropriate (18). This limitation highlights the necessity of implementing time-varying covariates in such analyses. Meanwhile, although the association between residential industrial air pollution exposure and disease risk has been reported, the modifying effects of age and exercise behavior on this association require further exploration (19). Research specifically targeting older adult populations remains particularly sparse. Therefore, survival analyses utilizing large-scale longitudinal cohort data are needed. Such analyses should incorporate time-varying parameters for industrial air pollution indicators and exercise behavior, thereby improving understanding of their interaction in relation to all-cause mortality risk in older adults.

In summary, this study aims to quantify the interaction effects between self-reported current exercise habits and county-level industrial air pollution indicators using Cox proportional hazards models applied to nationwide older adult survival cohort data. Specifically, we aim to determine whether county-level industrial air pollution indicators increase mortality risk by attenuating the association between self-reported current exercise habits and mortality. The findings will provide scientific evidence for developing exercise recommendations tailored to older adults residing in polluted regions.

## 2 Materials and methods

### 2.1 Study design and data sources

Data were obtained from the Chinese Longitudinal Healthy Longevity Survey (CLHLS) (20). The survey adopted a multistage stratified sampling method, covering 23 provinces, autonomous regions, and municipalities nationwide.

This study used longitudinal follow-up data from 2008 to 2014, including three waves (conducted in 2008–2009, 2011–2012, and 2014). The inclusion criteria required participants to be aged ≥ 65 years. A total of 16,954 older adults were enrolled. Extracted variables and their coding were as follows: (1) demographic characteristics: baseline age, sex (1 = male, 2 = female); (2) behavioral indicators: self-reported current exercise habits (0 = no, 1 = yes), smoking status (0 = non-smoker, 1 = smoker); (3) outcome event: all-cause mortality, with the year of death recorded. Survival time was determined as follows: trained interviewers confirmed the vital status of participants at each follow-up wave. For those who had died since the previous wave, the year of death was recorded, based on reports from family members or community informants. Survival time was calculated as the interval from the 2008 baseline to the recorded year of death or to the year of the last follow-up for censored cases, expressed in years. (4) Health status: measured by interviewer-rated overall health status on a 4-point ordinal scale (1 = surprisingly healthy, 2 = relatively healthy, 3 = moderately ill, and 4 = very ill, with higher scores indicating worse health status, and this rating was reassessed at each follow-up wave). This rating was assigned by trained interviewers following completion of the full structured questionnaire and on-site physical measurements, integrating information on self-rated health, cognitive function, activities of daily living and instrumental activities of daily living, physiological indicators (e.g., blood pressure, heart rate, vision, hearing), and chronic disease history. (5) Additional covariates: residence (1 = city, 2 = town, 3 = rural), body mass index (BMI, kg/m^2^), functional status (measured by ability to carry a 5-kg weight, 1 = able, 2 = a little difficult, 3 = unable to do so), and comorbidity count (sum of self-reported physician-diagnosed conditions from a list of 22 diseases; the specific conditions are listed in Supplementary Table 1).

To evaluate regional industrial air pollution burden, this study further integrated accompanying community tracking survey data (1998–2014) from CLHLS (21). These data were collected through a companion survey that gathered environmental indicators at the same follow-up waves as the main CLHLS survey, and linked to individual participants via their unique ID. For this study, we extracted participants’ indicators corresponding to the three follow-up waves of the main CLHLS survey (conducted in 2008–2009, 2011–2012, and 2014). These indicators represent county-level industrial air pollution indicators for participant residence locations, including: (1) soot emission (tons/year) and soot removal (tons/year); (2) SO_2_ emission (tons/year) and SO_2_ removal (tons/year). Additionally, county area (km^2^) was extracted for area-standardized sensitivity analyses. To improve regression coefficient interpretability, all county-level industrial air pollution indicators were standardized (converted to *Z*-scores).

The Peking University Biomedical Ethics Committee approved the CLHLS (ethics approval number: IRB00001052-13074). All methods were performed in accordance with relevant guidelines and regulations, and all participants provided written informed consent. This study obtained data use permission through the Peking University Open Research Data Platform.

### 2.2 Statistical analysis

#### 2.2.1 Survival data structure and covariate selection

Survival time data were analyzed using Cox proportional hazards models with time-varying covariates. The data were structured using a counting-process (start-stop) format. The time origin was set at the 2008 baseline survey, and survival time was calculated as described in Section 2.1. For participants who died between waves, the year of death was recorded at the subsequent survey and the event was assigned to the corresponding interval. Participants who were lost to follow-up between waves or remained alive at the end of the study period were treated as right-censored at their last confirmed vital status.

Among the covariates, sex and baseline age were treated as fixed, while self-reported current exercise habits, smoking status, health status, residence, BMI, functional status, and comorbidity count were treated as time-varying and updated at each follow-up wave. County-level industrial air pollution indicators were also treated as time-varying and matched to the long-format data by participants’ unique ID. For follow-up data, covariate values between two adjacent surveys were assumed to remain unchanged until the next survey, with each wave converted to a long-format data structure using SPSS 23.0 software. The proportional hazards assumption for all variables was tested using Schoenfeld residual plots. A horizontal band pattern of residuals without a systematic time trend was considered to satisfy the PH assumption.

Covariates were selected *a priori* based on established epidemiological evidence and biological plausibility. Sex and baseline age were included as fundamental demographic confounders (22). Given the high mean baseline age (86.8 years) in this cohort, baseline age was modeled with both linear and quadratic terms to allow nonlinearity. Smoking status was adjusted for its known relationship with mortality and respiratory health (23). Health status was included because it has been repeatedly identified as a strong predictor of survival among older adults (24, 25). Residence was included because urban–rural disparities in mortality have been well documented (26, 27). BMI was adjusted for its established association with mortality across diverse populations (28, 29). Functional status was included as a key indicator of physiological reserve, given its strong association with survival in older adults (30, 31). Comorbidity count was incorporated because it is a robust predictor of mortality in aging populations (32, 33). To assess potential multicollinearity among covariates, Spearman’s rank correlation coefficients were calculated using baseline (2008) data. As shown in Supplementary Tables 2, 3, correlations among covariates were low to modest, indicating no multicollinearity concerns.

#### 2.2.2 Cox proportional hazards models construction

**Figure 1:**
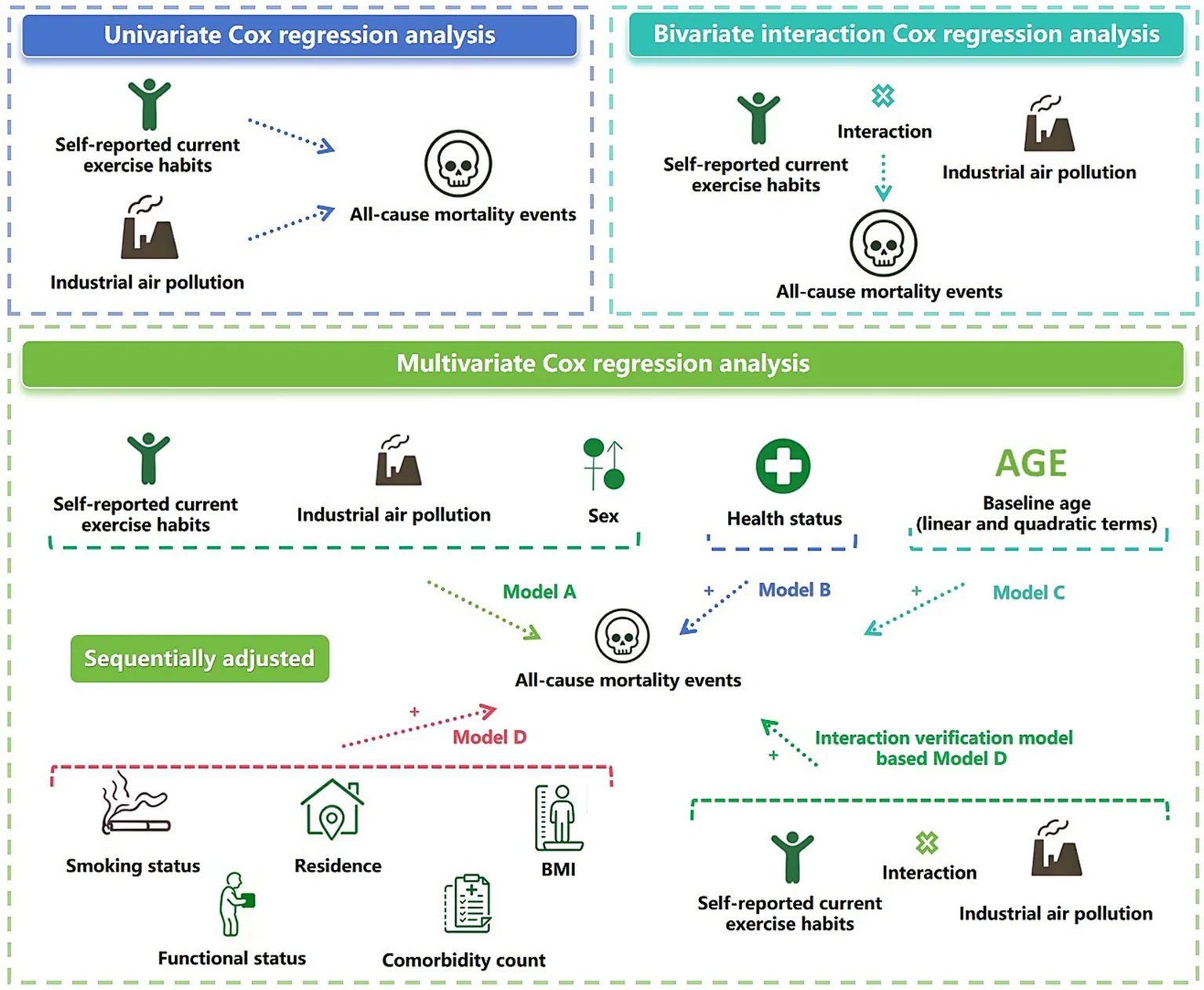
Logic diagram for Cox proportional hazards models construction.

Survival analysis was conducted through Cox proportional hazards models. Variable categorization and model construction strategies were as follows: (1) Core variables: self-reported current exercise habits, county-level industrial air pollution indicators; (2) Covariates: sex, health status (entered as a continuous variable using its ordinal 1–4 scale, with higher scores indicating worse health status), baseline age (linear and quadratic terms), smoking status, residence, BMI, functional status, and comorbidity count; (3) Cox model construction strategy: sequentially constructing ① univariate Cox models for core variables; ② Cox models incorporating core variable interaction terms; ③ multivariate Cox models including core variables, covariates, and the core variable interaction terms. A sequential adjustment method was used to construct multivariate Cox models as follows: model A (core variables and sex covariate), model B (model A and health status), model C (model B and baseline age with linear and quadratic terms), and fully adjusted model D (model C with smoking status, residence, BMI, functional status, and comorbidity count). An interaction verification model was also constructed, adding the interaction term between self-reported current exercise habits and the respective industrial air pollution indicator to model D. Each county-level industrial air pollution indicator was analyzed in a separate Cox model. Figure 1 summarizes the Cox proportional hazards model construction strategy. To account for geographic clustering, all models used province-clustered robust standard errors, as county-level geographic identifiers were not available in the CLHLS to protect participant privacy. For categorical variables, the reference categories were: no exercise habits (value = 0) for self-reported current exercise habits, male (value = 1) for sex, non-smoker (value = 0) for smoking status, and rural (value = 3) for residence. Other variables were entered as continuous variables.

Cox proportional hazards analysis was performed using StataMP-64 software. A forest plot of hazard ratios (HR) and 95% confidence intervals (CI) for each variable in the multivariate model was drawn using GraphPad Prism 8.0 software. Statistical significance was set at *α* = 0.05.

#### 2.2.3 Sensitivity analyses and power analysis

Four sensitivity analyses were conducted to evaluate the robustness of our findings. All sensitivity analyses used the same fully adjusted Model D specification: (1) A cumulative average model, replacing county-level industrial air pollution indicator values at each wave with the individual-specific cumulative mean up to each wave, to evaluate the impact of cumulative versus instantaneous indicator specification; (2) A last-observation-carried-forward (LOCF) model, carrying forward 2011 wave values to the 2014 wave (pollution data were incomplete for the 2014 follow-up wave in the CLHLS community tracking dataset); (3) An area-standardized model, in which county-level industrial air pollution indicators were standardized by county area (tons/km^2^) to account for variations in county size; (4) A reverse causation model, in which participants who died within the first 2 years of follow-up (2008–2010) were excluded.

Statistical power for interaction detection was estimated post-hoc using the non-central χ^2^ approximation for the Wald test of the interaction term (*df* = 1) in the Cox proportional hazards models. Calculations assumed a two-sided *α* = 0.05, with the observed sample size (*n* = 16,954), number of death events (*n* = 8,224), and baseline prevalence of self-reported exercise habits in this study (approximately 30%). Statistical power was estimated across a range of assumed interaction hazard ratios (HR = 1.10, 1.15 and 1.20).

## 3 Result

### 3.1 Baseline characteristics and survival time descriptive statistics

**Figure 2:**
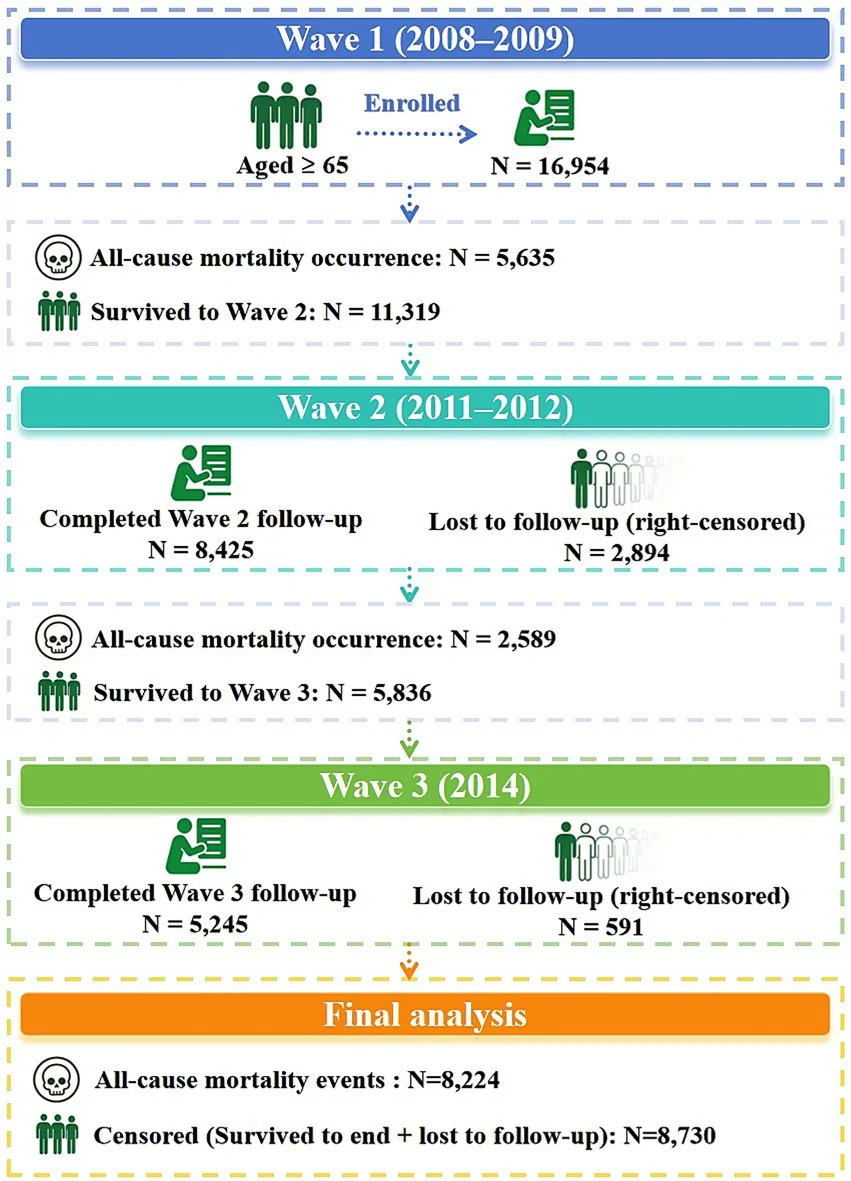
Participant flow diagram of the study cohort.

A total of 16,954 older adults aged 65 years and above were included in this study, comprising 7,252 males (42.77%) and 9,702 females (57.23%). The participant flow diagram is presented in Figure 2. During the 6-year follow-up period, 8,224 all-cause mortality events occurred. Table 1 presents the baseline characteristics, as well as survival status during follow-up. The minimum survival time was 0.0 years, corresponding to participants who died in the same year as the 2008 baseline survey. These participants (*n* = 807) were retained in the analysis to avoid selection bias. Participants were distributed across more than 800 county-level administrative units in 23 provinces. However, detailed county-level distribution data are not publicly available to protect participant privacy.

**Table 1:** Baseline characteristic distribution and survival time descriptive statistics.

Variable category	Variable name	Value
Baseline characteristics	Total sample size	16,954
	Age (years), Mean (SD)	86.8 (12.0)
	Sex, *n* (%)	
	Male	7,252 (42.77)
	Female	9,702 (57.23)
	Self-reported current exercise habits, *n* (%)	
	Yes	4,645 (27.40)
	No	12,308 (72.60)
	Missing	1 (0.01)
	Smoking status, *n* (%)	
	Smoker	2,966 (17.49)
	Non-smoker	13,988 (82.51)
	Residence, *n* (%)	
	City	3,351 (19.77)
	Town	3,310 (19.52)
	Rural	10,293 (60.71)
	Health status (1–4 scale), Mean (SD)	1.99 (0.68)
	BMI (kg/m^2^), Median (P_25_, P_75_)	19.84 (17.78, 22.27)
	Functional status (1–3 scale), Mean (SD)	1.82 (0.89)
	Comorbidity count, Median (P_25_, P_75_)	1 (0, 2)
	SO_2_ emission (×10^3^ tons/year), Median (P_25_, P_75_)	61.5 (43.9, 92.0)
	SO_2_ removal (×10^3^ tons/year), Median (P_25_, P_75_)	51.2 (20.2, 151.7)
	Soot emission (×10^3^ tons/year), Median (P_25_, P_75_)	21.5 (12.7, 37.1)
	Soot removal (×10^3^ tons/year), Median (P_25_, P_75_)	835.2 (353.6, 1684.5)
Survival status	Mortality events, *n* (%)	8,224 (48.51)
	Censored participants^a^, *n* (%)	8,730 (51.49)
	Survival time (years), Mean (SD)	4.3 (2.1)
	Minimum	0.0
	Maximum	6.0

SD: standard deviation. ^a^Censored includes participants who survived to the end of follow-up (*n* = 5,245) and those lost to follow-up (right-censored, *n* = 3,485). A survival time of 0.0 years indicates that the participant died in the same year as the 2008 baseline survey. These individuals were retained in the analysis to avoid selection bias.

### 3.2 Proportional hazards assumption test results

**Figure 3:**
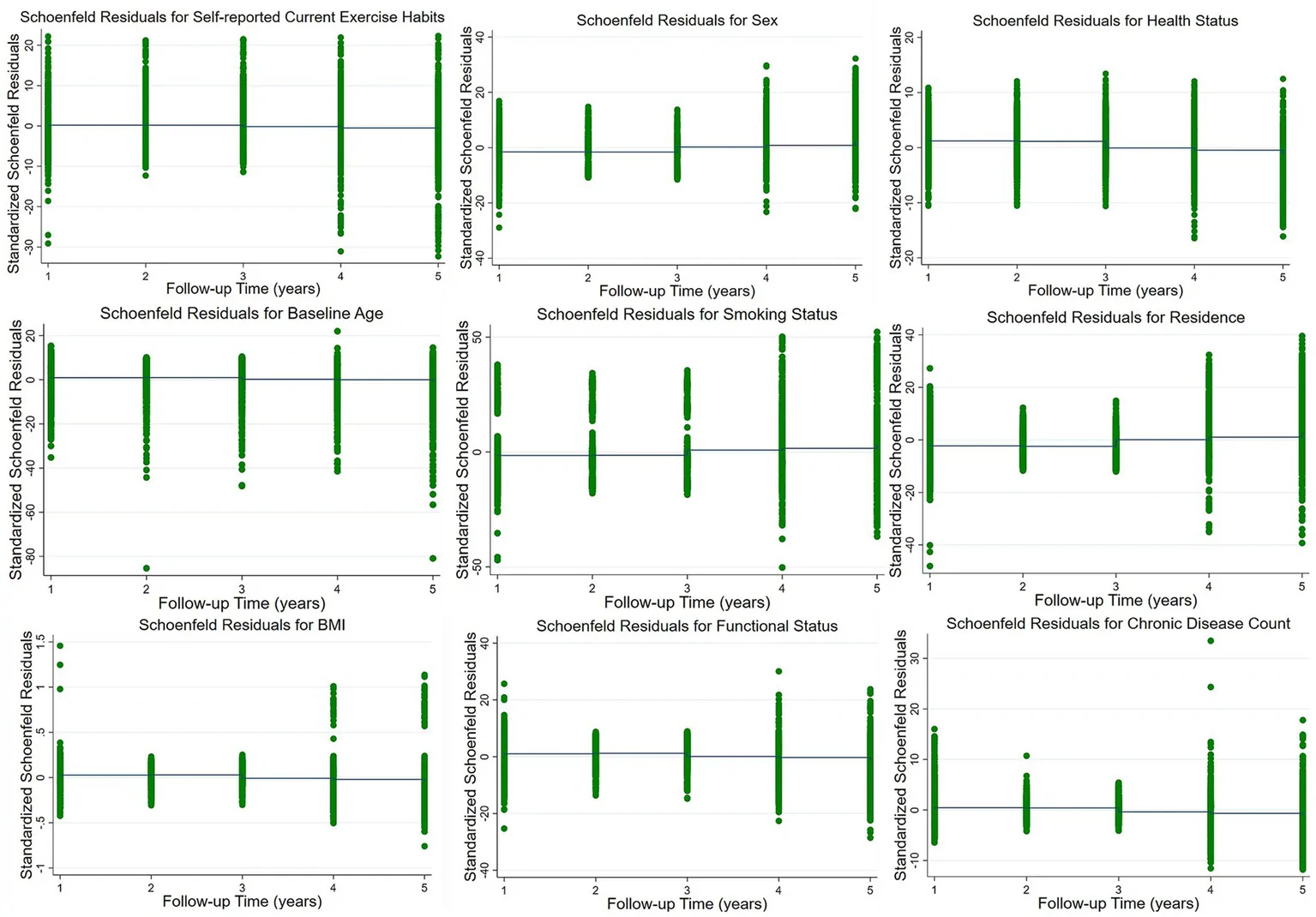
Schoenfeld residual plots for covariates. This figure presents the residual plots for all covariates except the county-level industrial air pollution indicators, based on the fully adjusted model with SO_2_ emission as the representative pollutant.

**Figure 4:**
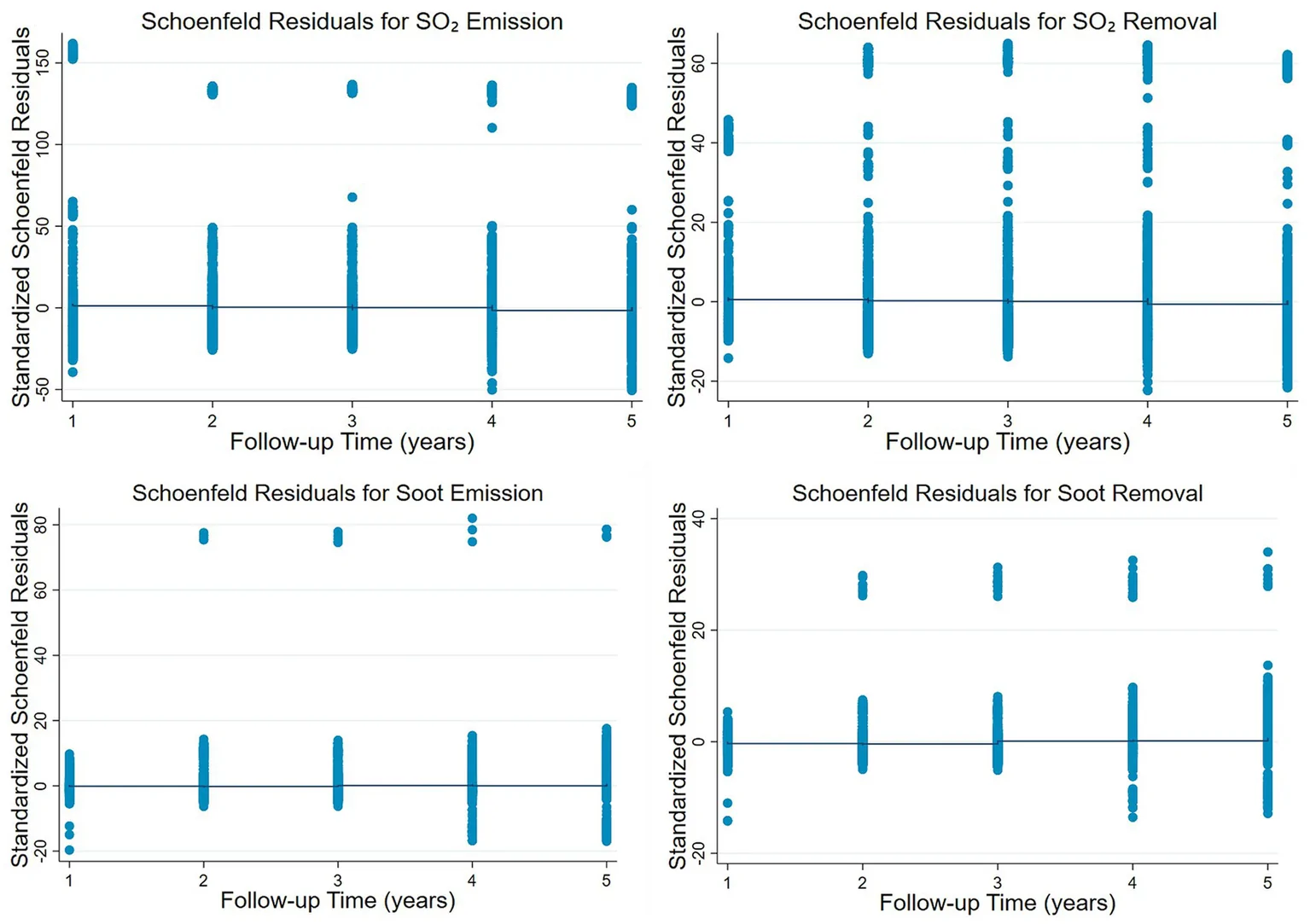
Schoenfeld residual plots for county-level industrial air pollution indicators. This figure presents the residual plots for each of the county-level industrial air pollution indicators, based on their respective fully adjusted models.

The proportional hazards assumption was assessed using Schoenfeld residual plots (Figures 3, 4). Figure 3 presents the residual plots for all covariates except the county-level industrial air pollution indicators, based on the fully adjusted model with SO_2_ emission as the representative pollutant. Figure 4 presents the residual plots for each of the county-level industrial air pollution indicators, based on their respective fully adjusted models. Formal tests of the proportional hazards assumption are provided in Supplementary Table 4. Although formal tests were statistically significant (*p* < 0.001), this is commonly observed in large-scale cohort studies due to high statistical power (34). Visual inspection of the Schoenfeld residual plots revealed horizontal band patterns with smoothed lines approximating horizontal trajectories without systematic temporal trends. Given the large sample size in this study, the proportional hazards assumptions were considered acceptable.

### 3.3 Univariate and two-way interaction cox regression analysis results

Univariate Cox regression analyses were performed for self-reported current exercise habits and for each of the four county-level industrial air pollution indicators separately. An interaction term between self-reported current exercise habits and each county-level industrial air pollution indicator was then added to the respective model. Results are shown in Table 2. Univariate analysis showed that self-reported current exercise habits were significantly associated with lower all-cause mortality risk in older adults (HR = 0.697, 95%CI: 0.643–0.757, *p* < 0.001), whereas main effects for all four county-level industrial air pollution indicators were not significant (*p* > 0.05). In the two-way interaction Cox regression models, all *p* values for the interaction terms between self-reported current exercise habits and the four county-level industrial air pollution indicators exceeded 0.05, suggesting that county-level industrial air pollution indicators did not significantly modify the association between self-reported current exercise habits and mortality risk in older adults.

**Table 2:** Univariate Cox regression and interaction analysis results.

Variable	HR	*p*	95% CI
Univariate Cox regression results			
Self-reported current exercise habits	0.697	<0.001	0.643−0.757
SO_2_ emission (per SD)	1.023	0.585	0.942−1.111
SO_2_ removal (per SD)	1.008	0.853	0.931−1.091
Soot emission (per SD)	1.008	0.657	0.975−1.042
Soot removal (per SD)	1.007	0.666	0.977−1.037
Two-way interaction Cox regression results			
Self-reported current exercise habits × Soot emission (per SD)	0.968	0.488	0.882−1.062
Self-reported current exercise habits × Soot removal (per SD)	1.011	0.661	0.962−1.062
Self-reported current exercise habits × SO_2_ emission (per SD)	0.967	0.161	0.922−1.014
Self-reported current exercise habits × SO_2_ removal (per SD)	0.981	0.105	0.958−1.004

SD, standard deviation. One SD for SO_2_ emission = 110.9 thousand tons/year; one SD for SO_2_ removal = 175.1 thousand tons/year; one SD for soot emission = 46.8 thousand tons/year; one SD for soot removal = 5,481.5 thousand tons/year. Reference categories: no exercise habits (value = 0) for self-reported current exercise habits, male (value = 1) for sex, non-smoker (value = 0) for smoking status, and rural (value = 3) for residence.

### 3.4 Multivariate cox regression analysis results

Given that SO_2_ emission represents a characteristic industrial air pollutant, this study employed SO_2_ emission as an example to illustrate multivariate Cox model sequential adjustment procedures in the main text, thereby controlling the number of tables in the main body. Results for other county-level industrial air pollution indicators are provided in Supplementary Tables 5–10.

Multivariate analysis results using SO_2_ emission as an example are shown in Table 3. After adjusting for sex (Model A), the HR for self-reported current exercise habits was 0.679 (*p* < 0.001). After further adjusting for health status (Model B), the HR for self-reported current exercise habits increased to 0.739 (*p* < 0.001). Each standard deviation increase in SO_2_ emission (110.9 thousand tons/year) was associated with a 3.1% increase in mortality risk (HR = 1.031, *p* = 0.413). After adding baseline age with linear and quadratic terms (Model C), self-reported current exercise habits remained significantly associated with lower mortality (HR = 0.807, *p* < 0.001), whereas the SO_2_ emission effect on mortality risk attenuated substantially from HR = 1.031 to HR = 1.014 (*p* = 0.752), suggesting that age was an important confounding factor. In the fully adjusted model (Model D), which additionally included smoking status, residence, BMI, functional status, and comorbidity count, self-reported current exercise habits remained significantly associated with lower mortality (HR = 0.835, *p* < 0.001), whereas the SO_2_ emission effect attenuated (HR = 1.001, *p* = 0.992). Similar patterns were observed for the other three pollution indicators (Supplementary Tables 5, 7, 9), with all pollutant main effects remaining non-significant in fully adjusted models.

**Table 3:** Multivariate Cox regression analysis results using SO_2_ emission as an example.

Model adjustment step	Exercise HR (95%CI)	*p*-value	SO_2_ emission (95%CI)	*p*-value
Model A: Exercise + SO_2_ emission (per SD) + sex	0.679 (0.619–0.745)	*p* < 0.001	1.033 (0.956–1.116)	*p* = 0.415
Model B: Model A + Health status	0.739 (0.673–0.812)	*p* < 0.001	1.031 (0.958–1.110)	*p* = 0.413
Model C: Model B + Baseline age (linear and quadratic terms)	0.807 (0.750–0.868)	*p* < 0.001	1.014 (0.933–1.102)	*p* = 0.752
Model D: Fully adjusted^a^	0.835 (0.773–0.902)	*p* < 0.001	1.001 (0.874–1.146)	*p* = 0.992
Interaction verification model: Model D + Self-reported current exercise habits×SO_2_ emission (per SD)	Interaction term HR: 0.970 (0.910–1.035), *p* = 0.355			

^a^Model D adjusted for sex, health status, baseline age (linear and quadratic terms), smoking, residence, BMI, functional status, and comorbidity count, with province-clustered robust standard errors. SD: standard deviation. One SD for SO_2_ emission = 110.9 thousand tons/year. Reference categories: no exercise habits (value = 0) for self-reported current exercise habits, male (value = 1) for sex, non-smoker (value = 0) for smoking status, and rural (value = 3) for residence.

The interaction verification model, which added the interaction term between self-reported current exercise habits and SO_2_ emission to Model D, showed a non-significant interaction effect (HR = 0.970, *p* = 0.355), indicating that county-level industrial air pollution did not significantly modify the association between self-reported current exercise habits and mortality among older adults. For the other three industrial air pollution indicators, the *p*-values for the interaction term were also non-significant (SO_2_ removal: HR = 0.994, *p* = 0.791; soot emission: HR = 0.979, *p* = 0.327; soot removal: HR = 0.984, *p* = 0.259; Supplementary Tables 5–10), further supporting the robustness of these findings.

**Figure 5:**
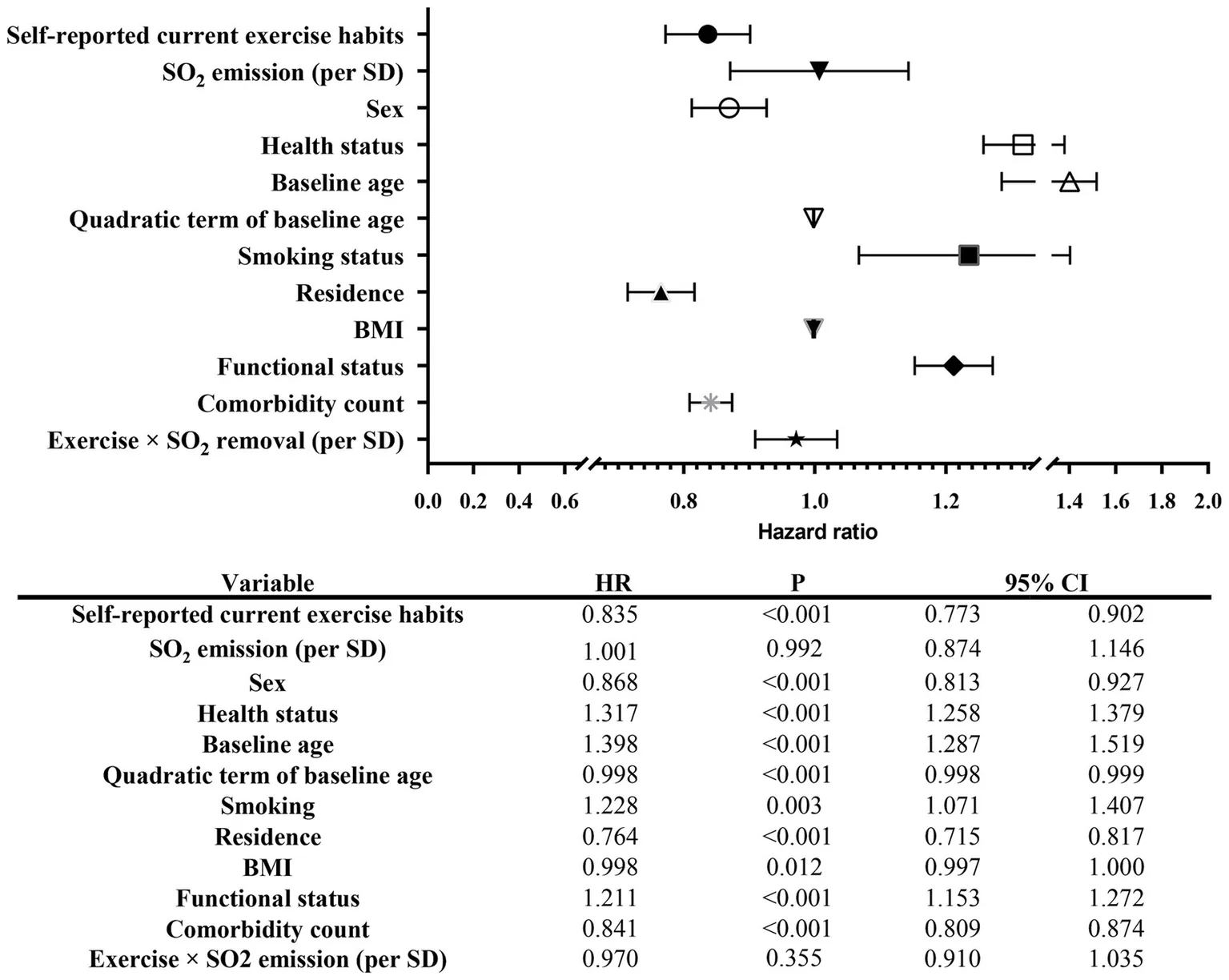
Forest plot of effect sizes from the full model (model D) using SO_2_ emission as an example. One SD for SO_2_ emission = 110.9 thousand tons/year. Baseline age was modeled with linear and quadratic terms (Wald joint test for both terms, *p* < 0.001).

Figure 5 shows a forest plot of effect sizes from the full model (Model D) using SO_2_ emission as an example, presenting the effect size and directions for self-reported current exercise habits, SO_2_ emission, and other covariates. Fully adjusted model results of the other three industrial air pollution indicators are provided in Supplementary Tables 6, 8, 10.

### 3.5 Sensitivity and statistical power analyses results

Sensitivity analyses confirmed the robustness of our primary findings (Table 4). In the cumulative average model, self-reported current exercise habits remained significantly associated with lower all-cause mortality (HR = 0.836, *p* < 0.001), and the interaction between self-reported current exercise habits and SO_2_ emission was non-significant (*p* = 0.372). In the LOCF model, which retained all 2014 observations by carrying forward 2011 pollution values, results were consistent with the primary analysis (HR = 0.836 for self-reported current exercise habits, *p* < 0.001; interaction *p* = 0.354). In the area-standardized model, the interaction between self-reported current exercise habits and SO_2_ emission was non-significant (*p* = 0.675). In the reverse causation analysis excluding participants who died within the first 2 years of follow-up, the association between self-reported current exercise habits and lower mortality remained significant (HR = 0.855, *p* < 0.001), and the interaction between self-reported current exercise habits and SO_2_ emission was also non-significant (*p* = 0.341). Sensitivity analyses for SO_2_ removal, soot emission, and soot removal yielded consistent results, with all interaction *p*-values remaining non-significant (Supplementary Table 11). These findings indicate that the conclusions are robust to alternative model specifications.

**Table 4:** Sensitivity analyses for the interaction between exercise and SO_2_ emission on all-cause mortality.

Sensitivity analysis strategy	Self-reported current exercise habits HR (95%CI)	*p*-value for interaction ^a^
Primary analysis (time-varying exposure)	0.835 (0.773–0.902)^*^	*p* = 0.355
Cumulative average model	0.836 (0.772–0.905)^*^	*p* = 0.372
LOCF model	0.836 (0.773–0.905)^*^	*p* = 0.354
Area-standardized model	0.840 (0.774–0.912)^*^	*p* = 0.675
Reverse causation model	0.855 (0.787–0.930)^*^	*p* = 0.341

^*^p < 0.001. ^a^Interaction term between self-reported current exercise habits and SO_2_ emission (per SD). One SD for SO_2_ emission = 110.9 thousand tons/year. All sensitivity analyses used the same fully adjusted Model D specification.

Post-hoc statistical power analysis indicated that this study had adequate statistical power to detect small to moderate interaction effects. As shown in Supplementary Table 12, with 16,954 participants and 8,224 mortality events, this study had 97.73% power to detect an interaction HR of 1.10 at a two-sided *α* = 0.05. Power exceeded 99.99% for larger effect sizes (HR ≥ 1.15). These results suggest that the non-significant interaction findings are unlikely to be attributable to insufficient statistical power.

## 4 Discussion

This study shows that self-reported current exercise habits are associated with lower all-cause mortality risk in Chinese older adults, with this association remaining robust after adjustment for county-level industrial air pollution indicators. The interaction between self-reported current exercise habits and county-level industrial air pollution indicators was not statistically significant.

Self-reported current exercise habits were consistently associated with lower all-cause mortality among Chinese older adults. Even after incorporating county-level industrial air pollution indicators, sex, health status, baseline age, smoking status, and additional covariates (residence, BMI, functional status, and comorbidity count) within Cox models, self-reported current exercise habits remained significantly associated with lower all-cause mortality (Model D: HR = 0.835, *p* < 0.001). This finding aligns with multiple studies (12, 16, 17, 35). Relevant research indicates that exposure to air pollution (such as NO_2_) does not attenuate the protective effect of exercise on mortality risk (12, 16, 17), suggesting that the health benefits linked to exercise are environmentally adaptable. One investigation involving older adult populations in Hong Kong demonstrated that habitual PA and long-term PM_2.5_ exposure exhibited inverse associations regarding dementia events, with PA benefits remaining significant across varying pollution exposure levels (35). Andersen et al. (17) also noted that short-term exposure to air pollution during exercise produces acute cardiovascular responses such as vascular injury, arterial stiffness, and reduced blood flow, but these injuries appear transient and reversible, and do not attenuate the long-term survival benefit associated with exercise. Beyond the biological plausibility discussed above, the robustness of this finding was further supported by sensitivity analyses. Regardless of whether county-level industrial air pollution indicators were specified as cumulative averages over time or via the LOCF approach, the association between self-reported current exercise habits and lower mortality remained significant. In addition, sensitivity analyses using area-standardized model and excluding participants who died within the first 2 years of follow-up also yielded consistent results, further supporting the robustness of our findings. These consistent results across alternative indicator specifications are compatible with the interpretation that the survival benefit of self-reported current exercise habits might not be meaningfully modified by county-level industrial air pollution indicators at the population level.

Additionally, complex associations may exist between health status and self-reported current exercise habits. The inclusion of health status in the model resulted in an attenuation of the HR values for self-reported current exercise habits (from 0.679 to 0.739), suggesting that health status may partially explain the observed association between self-reported current exercise habits and mortality. Health status was strongly associated with mortality risk in older adults (Model D: HR = 1.317, *p* < 0.001), and self-reported current exercise habits may influence mortality through improvements in overall health. However, formal mediation analysis is needed to confirm this pathway. Air pollution exposure may impair body function through enhanced oxidative stress and inflammatory responses (36, 37). In contrast, exercise may partially counteract these effects by enhancing immune function, reducing systemic inflammation, and regulating metabolic homeostasis (38–40), thereby improving overall health and indirectly reducing mortality risk. The association between exercise and mortality in the context of air pollution involves dynamic balance across multiple physiological systems. In the cardiovascular system, exercise improves function by increasing cardiac output (41), improving vascular endothelial function (42), and releasing myogenic factors that inhibit chronic inflammation (43). Regarding the immune system, exercise enhances T-cell diversity (38), reduces adipose tissue-derived inflammatory factors (44), and enhances natural killer cell activity and tumor suppressor secretion (44, 45), effectively lowering infection risk. In the nervous system, post-exercise clusterin can inhibit neuroinflammation and reduce the risk of cognitive decline (46). Moreover, older adults residing within polluted environments are more prone to depression. The release of endorphins and other hormones during exercise lowers cortisol levels (47), effectively reducing depression risk in older adults. In summary, self-reported current exercise habits were associated with improvements across multiple health dimensions, which may contribute to the observed lower mortality risk. However, the specific pathways through which overall health status influences the relationship between exercise and mortality remain unclear. Future studies need to combine longitudinal data to further explore whether health indicators (including inflammatory factors, lung function, and mental health status) mediate the relationship between exercise and damage from air pollution exposure.

Previous studies have suggested that SO_2_ exposure may be associated with adverse health outcomes (11, 48). However, in our fully adjusted models, none of the four county-level industrial air pollution indicators showed significant associations with mortality. For SO_2_ emission, the effect was substantially attenuated after adjustment for covariates (Model D: HR = 1.001, *p* = 0.992), suggesting that the weak association observed in univariate analysis was largely explained by confounding factors, particularly age and additional covariates (residence, BMI, functional status, and comorbidity count). Failure to adjust for these covariates in the model could overestimate the negative effect of air pollution. Nevertheless, the long-term cumulative effect of SO_2_ should not be ignored, requiring continued emphasis on chronic industrial emission control. Previous studies have found that the synergistic effect of multi-pollutant exposure (including SO_2_, PM_10_, and NO_2_) may increase the risk of various diseases such as cardiovascular disease, metabolic syndrome, and sarcopenia, particularly in older adults and regions with severe long-term industrial pollution (49–52). A multi-city study in China showed that SO_2_ exposure was significantly associated with acute myocardial infarction hospital admissions (48). Furthermore, certain populations (e.g., physically active individuals and overweight individuals) show greater sensitivity to SO_2_ exposure (11, 50, 53), which may induce health damage through mechanisms including systemic inflammation and oxidative stress (11). Therefore, while the present study did not find a statistically significant association between county-level SO_2_ emission indicators and all-cause mortality after full covariate adjustment, the broader epidemiological evidence suggests that continued attention to industrial SO_2_ emission control remains warranted. Attention should be paid to the independent effect of SO_2_ in multi-pollutant exposure models. Previous studies indicate that the effect of SO_2_ on mortality is not confounded by other pollutant types (54), and that strengthening control over SO_2_ exposure may help extend life expectancy (55). Future pollution control strategies might be optimized based on regional energy structures (including coal dependency status) to reduce the long-term burden of industrial air pollution exposure on cardiometabolic health (56).

Several covariates included in the fully adjusted model were significantly associated with mortality. Notably, comorbidity count showed a counterintuitive inverse association with mortality. Using the fully adjusted model based on the SO_2_ emission indicator as an example, comorbidity count was inversely associated with mortality (Model D: HR = 0.841, *p* < 0.001). While multimorbidity is a well-established risk factor for mortality in general older populations (32, 57), this unexpected finding may be explained by the unique characteristics of our study population (mean age 86.8 years). Studies have shown that the predictive value of simple disease counts appears to be attenuated in extreme old age, potentially because the risk of mortality is not determined by the number of diseases themselves, but by whether these diseases lead to functional decline and frailty (58). Indeed, frailty has been shown to mediate the association between multimorbidity and mortality (58). In line with this, functional status, measured by the ability to carry 5 kg weight, was strongly associated with mortality in the fully adjusted model (HR = 1.211, *p* < 0.001), consistent with previous findings that impaired physical function and sarcopenia are robust predictors of adverse outcomes in older populations (30, 31). Similarly, health status also emerged as a significant predictor (Model D: HR = 1.317, *p* < 0.001), which was assessed by integrating information on self-rated health, cognitive function, activities of daily living and instrumental activities of daily living, physiological indicators. The above results suggest that, compared to simply focusing on the number of comorbidities, functional status and overall health may warrant greater attention in older adults. The inverse association between comorbidity count and mortality may also reflect survival selection bias, whereby individuals who reach extreme old age represent a selected group that has survived the highest-risk disease combinations (59). In line with this, baseline age showed a non-linear association with mortality in the fully adjusted model (joint test *p* < 0.001), with the rate of mortality increase attenuating at the oldest ages, which may reflect the selective survival of the most robust individuals. In addition, smoking remained a significant risk factor (Model D: HR = 1.228, *p* = 0.003), confirming its well-established detrimental effect on survival at advanced ages (60). Residence also emerged as a significant predictor, with urban residents showing lower mortality compared with their rural counterparts (Model D: HR = 0.764, *p* < 0.001), consistent with documented urban–rural health disparities in China (26, 61). BMI showed a modest but statistically significant inverse association with mortality (Model D: HR = 0.998, *p* = 0.012), which may reflect the complex and non-linear relationship between BMI and survival in older adults, where both low and extremely high BMI have been associated with increased mortality risk (28, 29). Similar patterns were observed in the fully adjusted models for other pollution indicators (Supplementary Tables 6, 8, 10). Collectively, these findings underscore the importance of accounting for multiple dimensions of health, including physical function, disease burden, socioeconomic context, and nutritional status, when examining the association between exercise and mortality in older populations. Future studies are necessary to further explore the mechanism of the association between comorbidity and mortality among older adults, especially whether there is a potential mediating effect of functional decline and frailty, as well as the changing role of lifestyle factors such as exercise.

In the multivariate Cox regression models, the interactions between self-reported current exercise habits and each of the four industrial air pollution indicators did not reach statistical significance. This was further supported by sensitivity analyses, in which both cumulative average and LOCF specifications yielded non-significant interactions. Moreover, post-hoc statistical power analysis confirmed that this study was sufficiently powered to detect a clinically relevant interaction effect (see Supplementary Table 12), reducing the possibility that the non-significant interaction findings stem from insufficient statistical power. Several methodological considerations should be noted when interpreting these findings. First, exposure misclassification may have attenuated the interaction estimates, as county-level industrial air pollution indicators do not capture individual-level spatial variability or activity patterns. Previous studies have shown that using predicted concentrations can introduce random exposure misclassification that attenuates health effect estimates (62), and that residence-only exposure assignment may weaken the association between pollutant and disease compared with activity-weighted estimates (63). Second, residual confounding from unmeasured factors cannot be entirely ruled out, particularly indoor air pollution from household cooking and heating, and traffic-related pollution. Cumulative biomass fuel use (per 50-h increase) has been associated with higher mortality risk in Chinese adults (HR = 1.18 for all-cause mortality) (64), and adjustment for road density has been shown to significantly strengthen NO_2_ mortality associations (65). Although we adjusted for smoking status (which partially captures indoor combustion sources), comprehensive adjustment for these factors was precluded by data availability. Nevertheless, the consistent non-significance across primary and sensitivity analyses supports the interpretation that the association between self-reported current exercise habits and mortality in older adults is not significantly affected by county-level industrial air pollution indicators. However, acute physiological effects warrant continued attention. For example, acute respiratory system damage from SO_2_ exposure may limit the improvement in lung function during exercise (9, 66). Moreover, different pollutant types may have different or interactive effects on health (67–69). The gaseous nature of SO_2_ may directly irritate respiratory tract mucosa, exerting a direct effect on pneumonia risk (66). In contrast, PM_2.5_, with its small particle size and strong penetrability, more readily affects health through oxidative stress and epigenetic regulation (70, 71), inducing autonomic nervous system dysfunction and vascular dysfunction that may attenuate the cardiovascular protection from PA (9, 70). PM_2.5_ may also interact with PA level to influence cancer risk (19). Due to limitations in the original data, this study could not analyze interactions between exercise and other air pollution exposure types such as PM_2.5_. Therefore, heterogeneity in pollutant indicator type may have contributed to the non-significant interaction effects observed in this study. Future studies should use multi-pollutant exposure interaction models to analyze dose–response relationships between different pollutant types (e.g., PM_2.5_ and SO_2_) and the protective effect of exercise.

This study provides evidence supporting non-pharmacological exercise interventions in public health practice. Even in areas with industrial air pollution, older adults should be encouraged to maintain habitual exercise behavior. Public health strategies might integrate industrial SO_2_ emission control with exercise promotion for older adults, potentially contributing to lower all-cause mortality risk.

### 4.1 Limitations and prospects

Several limitations of this study should be acknowledged. Current exercise habits were self-reported and measured as a binary variable, without more detailed parameters such as exercise intensity, frequency, or type. This restricts deeper analysis of the relationships among exercise, industrial air pollution exposure, and all-cause mortality. In addition, the questionnaire did not distinguish between indoor and outdoor physical activities, nor did it collect consistent information on activity location across follow-up waves during 2008–2014, which precluded separate analyses by exercise setting. The observed association between self-reported current exercise habits and mortality in this study likely reflects a combination of indoor and outdoor activities, but the relative contributions and potential differential effects of pollution indicator levels by activity location could not be determined. Future studies could combine wearable devices (e.g., accelerometers) with questionnaires to more precisely record specific parameters of exercise behavior and activity location, thereby more accurately assessing these relationships.

Regarding pollution indicator assessment, industrial air pollution indicators were measured at the county level from the CLHLS community tracking dataset. This spatial and temporal resolution is constrained by the original survey design and does not reflect finer-scale variations or shorter-term fluctuations. Attempts to incorporate higher-resolution pollution data were precluded by the lack of precise residential address information in the CLHLS. To address potential indicator measurement imprecision arising from both coarse resolution and incomplete 2014 pollution data, this study conducted sensitivity analyses using cumulative average and LOCF specifications. These analyses produced results consistent with the primary analysis (Table 4), partly addressing concerns about indicator measurement precision. Nevertheless, these analyses do not substitute for higher-resolution exposure data, which remains a priority for future research.

Additionally, although adjustment for health status attenuated the association between self-reported current exercise habits and all-cause mortality, the specific mechanism requires further validation. Targeted longitudinal follow-up studies are recommended, incorporating blood biomarkers (e.g., inflammatory factors and oxidative stress indicators) and objective physiological measures such as lung function, to further explore how health status may influence the association between exercise and mortality in the context of industrial air pollution. Despite these limitations, the findings of this study provide preliminary evidence for the health benefit associated with exercise behavior in areas with industrial emissions. To advance older adult exercise, health, and environmental governance policy, future investigations should employ more refined exposure measurement, more comprehensive confounding control, and more systematic exploration of mechanisms. In particular, the potential mediating role of functional decline and frailty in the association between multimorbidity and mortality in extreme old age warrants further investigation through longitudinal studies with repeated measures of both disease burden and physical function.

## 5 Conclusion

Self-reported current exercise habits were consistently associated with lower all-cause mortality among Chinese older adults, and this association remained robust after adjustment for covariates, including county-level industrial air pollution indicators. No statistically significant multiplicative interaction was detected between exercise habits and any of the four pollution indicators. Nevertheless, continued attention to chronic industrial air pollution control remains warranted. Future studies need to further investigate whether and how comprehensive health indicators, including physical function, disease burden, and lifestyle factors, may influence the association between exercise and mortality. Studies should also employ multi-pollutant models to analyze dose–response relationships between different air pollutant types and mortality. This study provides preliminary evidence that promoting habitual exercise behavior among older adults may be beneficial even in areas with industrial emissions.

## Data Availability

Publicly available datasets were analyzed in this study. This data can be found at: https://opendata.pku.edu.cn/dataset.xhtml?persistentId=doi:10.18170/DVN/WBO7LK.
