# Supplementary material for: Exercise is associated with lower all-cause mortality among older adults: a national cohort survival analysis with county-level industrial air pollution indicators
Source: Front Public Health. 2026 Sep 4;14:1894835. doi: 10.3389/fpubh.2026.1894835 (PMC13541438; doi:10.3389/fpubh.2026.1894835)
Supplement: Supplementary file 1 [file Table_1.DOCX]

**Supplementary Tables**

**Supplementary Table 1 List of 22 self-reported physician-diagnosed conditions included in the comorbidity count**

| **No.** | **Condition** |
| --- | --- |
| 1 | Hypertension |
| 2 | Diabetes mellitus |
| 3 | Heart disease |
| 4 | Stroke or cerebrovascular disease |
| 5 | Bronchitis, emphysema, pneumonia, or asthma |
| 6 | Tuberculosis |
| 7 | Cataract |
| 8 | Glaucoma |
| 9 | Cancer (malignant tumor) |
| 10 | Prostate tumor |
| 11 | Gastric or duodenal ulcer |
| 12 | Parkinson's disease |
| 13 | Bedsore (pressure ulcer) |
| 14 | Arthritis |
| 15 | Dementia |
| 16 | Epilepsy |
| 17 | Cholecystitis or cholelithiasis |
| 18 | Blood disease (hematological disorder) |
| 19 | Chronic nephritis |
| 20 | Galactophore disease (breast disease) |
| 21 | Uterine tumor |
| 22 | Hepatitis |

Note: Conditions were derived from the CLHLS standard disease module. Only responses coded as 1 (physician-diagnosed presence) were counted as positive. The comorbidity count represents the sum of these 22 diseases indicators.

**Supplementary Table 2 Spearman Correlation Matrix (*r* values) of Individual-Level Covariates**

| **Variable** | **Self-reported current exercise habits** | **Sex** | **Baseline age** | **Smoking status** | **Health status** | **Residence** | **BMI** | **Functional status** | **Comorbidity count** |
| --- | --- | --- | --- | --- | --- | --- | --- | --- | --- |
| Self-reported current exercise habits | 1.000 |  |  |  |  |  |  |  |  |
| Sex | -0.146 | 1.000 |  |  |  |  |  |  |  |
| Baseline age | -0.201 | 0.239 | 1.000 |  |  |  |  |  |  |
| Smoking status | 0.065 | -0.361 | -0.199 | 1.000 |  |  |  |  |  |
| Health status | -0.204 | 0.131 | 0.261 | -0.132 | 1.000 |  |  |  |  |
| Residence | -0.222 | 0.014 | -0.011 | 0.045 | 0.024 | 1.000 |  |  |  |
| BMI | 0.151 | -0.147 | -0.313 | 0.058 | -0.202 | -0.097 | 1.000 |  |  |
| Functional status | -0.262 | 0.274 | 0.568 | -0.197 | 0.414 | -0.029 | -0.250 | 1.000 |  |
| Comorbidity count | 0.078 | -0.003 | -0.069 | -0.059 | 0.197 | -0.155 | 0.056 | 0.070 | 1.000 |

**Supplementary Table 3 Spearman Correlations Between County-Level Industrial Air Pollution Indicators and Individual-Level Covariates**

| **Variable** | **SO_2_ emission**  **（*r* values）** | **SO_2_ removal**  **（*r* values）** | **Soot emission**  **（*r* values）** | **Soot removal**  **（*r* values）** |
| --- | --- | --- | --- | --- |
| Self-reported current exercise habits | 0.032 | 0.033 | 0.069 | 0.061 |
| Sex | -0.002 | -0.001 | -0.004 | 0.002 |
| Baseline age | -0.007 | -0.024 | 0.017 | 0.007 |
| Smoking status | -0.004 | 0.002 | -0.001 | -0.015 |
| Health status | -0.019 | -0.043 | -0.001 | -0.024 |
| Residence | -0.168 | -0.091 | -0.194 | -0.194 |
| BMI | 0.097 | 0.097 | 0.093 | 0.150 |
| Functional status | 0.011 | -0.001 | 0.009 | 0.036 |
| Comorbidity count | 0.039 | 0.033 | 0.097 | 0.080 |

Note: Industrial air pollution indicators are county-level totals (tons/year).

**Supplementary Table 4 Formal Tests of the Proportional Hazards Assumption for Each Fully Adjusted Model**

| **Variable** | **SO_2_ emission’s fully adjusted model (χ²)** | **SO_2_ removal’s fully adjusted model (χ²)** | **Soot emission’s fully adjusted model (χ²)** | **Soot removal’s fully adjusted model (χ²)** |
| --- | --- | --- | --- | --- |
| Global test (d*f*=11) | 16719.53^*^ | 17067.42^*^ | 15914.30^*^ | 11402.68^*^ |
| Self-reported current exercise habits (d*f*=1) | 135.25^*^ | 406.79^*^ | 231.08^*^ | 84.54^*^ |
| County-level industrial air pollution indicator (d*f*=1) | 492.96^*^ | 284.44^*^ | 124.71^*^ | 1068.66^*^ |
| Sex (d*f*=1) | 3955.56^*^ | 4486.92^*^ | 3713.90^*^ | 2695.18^*^ |
| Health status (d*f*=1) | 4005.46^*^ | 4096.07^*^ | 3455.60^*^ | 3748.30^*^ |
| Baseline age (d*f*=1) | 374.83^*^ | 489.50^*^ | 281.64^*^ | 199.72^*^ |
| Smoking status (d*f*=1) | 1420.65^*^ | 2091.65^*^ | 1408.53^*^ | 943.56^*^ |
| Residence (d*f*=1) | 7801.78^*^ | 8501.55^*^ | 7866.22^*^ | 3283.60^*^ |
| BMI (d*f*=1) | 5073.80^*^ | 4886.18^*^ | 4196.39^*^ | 2622.00^*^ |
| Functional status (d*f*=1) | 2473.12^*^ | 3019.87^*^ | 2813.68^*^ | 385.26^*^ |
| Comorbidity count (d*f*=1) | 2466.83^*^ | 2691.77^*^ | 2805.33^*^ | 1331.84^*^ |

Note: ^*^*P*<0.001. Although formal tests were statistically significant (*P*<0.001), this is commonly observed in large-scale cohort studies due to high statistical power. Visual inspection of Schoenfeld residual plots (Figures 3 and 4) revealed horizontal band patterns with smoothed lines approximating horizontal trajectories and no systematic temporal trends. Given the large sample size in this study, the proportional hazards assumptions were considered acceptable.

**Supplementary Table 5 Multivariate Cox Regression Analysis Results using SO_2_ Removal**

| **Model Adjustment Step** | **Exercise HR（95%CI）** | ***P*-value** | **SO_2_** **removal（95%CI）** | ***P*-value** |
| --- | --- | --- | --- | --- |
| **Model A:** Exercise + SO_2_ removal (per SD) + sex | 0.670(0.609-0.736) | *P*<0.001 | 1.014(0.938-1.097) | *P*=0.718 |
| **Model B:** Model A + Health status | 0.727(0.661-0.799) | *P*<0.001 | 1.015(0.945-1.091) | *P*=0.685 |
| **Model C:** Model B + Baseline age (linear and quadratic terms) | 0.793(0.740-0.851) | *P*<0.001 | 1.016(0.958-1.079) | *P*=0.593 |
| **Model D:** Fully adjusted ^a^ | 0.821(0.759-0.889) | *P*<0.001 | 1.016(0.932-1.108) | *P*=0.716 |
| **Interaction verification model:** Model D + Self-reported current exercise habits×SO_2_ removal (per SD) | Interaction term HR: 0.994(0.954-1.037), *P*=0.791 | | | |

Note: ^a^ Model D adjusted for sex, health status, baseline age (linear and quadratic terms), smoking, residence, BMI, functional status, and comorbidity count, with province-clustered robust standard errors. SD: standard deviation. One SD for SO_2_ removal=175.1 thousand tons/year. Reference categories: no exercise habits (value = 0) for self-reported current exercise habits, male (value =1) for sex, non-smoker (value = 0) for smoking status, and rural (value =3) for residence.

**Supplementary Table 6 Multivariate Cox Regression Analysis Results for SO_2_ Removal (Fully Adjusted Model)**

| **Variable** | **HR** | ***P*-value** | **95% CI** | |
| --- | --- | --- | --- | --- |
| Self-reported current exercise habits | 0.821 | <0.001 | 0.759 | 0.889 |
| SO_2_ removal (per SD) | 1.016 | 0.716 | 0.932 | 1.108 |
| Sex | 0.875 | <0.001 | 0.821 | 0.933 |
| Health status | 1.310 | <0.001 | 1.246 | 1.376 |
| Baseline age | 1.393 | <0.001 | 1.275 | 1.521 |
| Quadratic term of baseline age | 0.998 | <0.001 | 0.998 | 0.999 |
| Baseline age (linear and quadratic terms) ^a^ | / | <0.001 | / | / |
| Smoking status | 1.231 | 0.002 | 1.079 | 1.405 |
| Residence | 0.766 | <0.001 | 0.714 | 0.821 |
| BMI | 0.998 | 0.011 | 0.997 | 1.000 |
| Functional status | 1.210 | <0.001 | 1.150 | 1.273 |
| Comorbidity count | 0.836 | <0.001 | 0.805 | 0.869 |
| Self-reported current exercise habits × SO_2_ removal (per SD) | 0.994 | 0.791 | 0.954 | 1.037 |

Note: ^a^ Baseline age was modeled with linear and quadratic terms (Wald joint test for both terms). SD: standard deviation. One SD for SO_2_ removal=175.1 thousand tons/year. Reference categories: no exercise habits (value = 0) for self-reported current exercise habits, male (value =1) for sex, non-smoker (value = 0) for smoking status, and rural (value =3) for residence.

**Supplementary Table 7 Multivariate Cox Regression Analysis Results using Soot Emission**

| **Model Adjustment Step** | **Exercise HR（95%CI）** | ***P*-value** | **Soot emission（95%CI）** | ***P*-value** |
| --- | --- | --- | --- | --- |
| **Model A:** Exercise + soot emission (per SD) + sex | 0.679(0.618-0.746) | *P*<0.001 | 1.013(0.981-1.047) | *P*=0.427 |
| **Model B:** Model A + Health status | 0.740(0.672-0.815) | *P*<0.001 | 1.011(0.980-1.044) | *P*=0.487 |
| **Model C:** Model B + Baseline age (linear and quadratic terms) | 0.807(0.749-0.870) | *P*<0.001 | 1.009(0.964-1.055) | *P*=0.482 |
| **Model D:** Fully adjusted ^a^ | 0.834(0.769-0.904) | *P*<0.001 | 1.009(0.964-1.055) | *P*=0.711 |
| **Interaction verification model:** Model D + Self-reported current exercise habits×Soot emission (per SD) | Interaction term HR: 0.979(0.940-1.021), *P*=0.327 | | | |

Note: ^a^ Model D adjusted for sex, health status, baseline age (linear and quadratic terms), smoking, residence, BMI, functional status, and comorbidity count, with province-clustered robust standard errors. SD: standard deviation. One SD for soot emission=46.8 thousand tons/year. Reference categories: no exercise habits (value = 0) for self-reported current exercise habits, male (value =1) for sex, non-smoker (value = 0) for smoking status, and rural (value =3) for residence.

**Supplementary Table 8 Multivariate Cox Regression Analysis Results for Soot Emission (Fully Adjusted Model)**

| **Variable** | **HR** | ***P*-value** | **95% CI** | |
| --- | --- | --- | --- | --- |
| Self-reported current exercise habits | 0.834 | <0.001 | 0.769 | 0.904 |
| Soot emission (per SD) | 1.009 | 0.711 | 0.964 | 1.055 |
| Sex | 0.868 | <0.001 | 0.813 | 0.927 |
| Health status | 1.315 | <0.001 | 1.256 | 1.377 |
| Baseline age | 1.396 | <0.001 | 1.283 | 1.518 |
| Quadratic term of baseline age | 0.998 | <0.001 | 0.998 | 0.999 |
| Baseline age (linear and quadratic terms) ^a^ | / | <0.001 | / | / |
| Smoking status | 1.227 | 0.003 | 1.071 | 1.406 |
| Residence | 0.764 | <0.001 | 0.714 | 0.817 |
| BMI | 0.998 | 0.004 | 0.997 | 0.999 |
| Functional status | 1.212 | <0.001 | 1.153 | 1.274 |
| Comorbidity count | 0.841 | <0.001 | 0.808 | 0.875 |
| Self-reported current exercise habits ×Soot emission (per SD) | 0.979 | 0.327 | 0.940 | 1.021 |

Note: ^a^ Baseline age was modeled with linear and quadratic terms (Wald joint test for both terms). SD: standard deviation. One SD for soot emission=46.8 thousand tons/year. Reference categories: no exercise habits (value = 0) for self-reported current exercise habits, male (value =1) for sex, non-smoker (value = 0) for smoking status, and rural (value =3) for residence.

**Supplementary Table 9 Multivariate Cox Regression Analysis Results using Soot removal**

| **Model Adjustment Step** | **Exercise HR（95%CI）** | ***P*-value** | **Soot removal（95%CI）** | ***P*-value** |
| --- | --- | --- | --- | --- |
| **Model A:** Exercise + Soot removal (per SD) + sex | 0.693(0.631-0.761) | *P*<0.001 | 1.015(0.985-1.046) | *P*=0.332 |
| **Model B:** Model A + Health status | 0.754(0.685-0.829) | *P*<0.001 | 1.020(0.992-1.049) | *P*=0.156 |
| **Model C:** Model B + Baseline age (linear and quadratic terms) | 0.821(0.762-0.884) | *P*<0.001 | 1.016(0.992-1.040) | *P*=0.200 |
| **Model D:** Fully adjusted ^a^ | 0.857(0.795-0.925) | *P*<0.001 | 1.002(0.966-1.039) | *P*=0.922 |
| **Interaction verification model:** Model D + Self-reported current exercise habits×Soot removal (per SD) | Interaction term HR: 0.984(0.955-1.021), *P*=0.259 | | | |

Note: ^a^ Model D adjusted for sex, health status, baseline age (linear and quadratic terms), smoking, residence, BMI, functional status, and comorbidity count, with province-clustered robust standard errors. SD: standard deviation. One SD for soot removal=5,481.5 thousand tons/year. Reference categories: no exercise habits (value = 0) for self-reported current exercise habits, male (value =1) for sex, non-smoker (value = 0) for smoking status, and rural (value =3) for residence.

**Supplementary Table 10 Multivariate Cox Regression Analysis Results for Soot Removal (Fully Adjusted Model)**

| **Variable** | **HR** | ***P*-value** | **95% CI** | |
| --- | --- | --- | --- | --- |
| Self-reported current exercise habits | 0.857 | <0.001 | 0.795 | 0.925 |
| Soot removal (per SD) | 1.002 | 0.922 | 0.966 | 1.039 |
| Sex | 0.854 | <0.001 | 0.797 | 0.914 |
| Health status | 1.335 | <0.001 | 1.276 | 1.397 |
| Baseline age | 1.390 | <0.001 | 1.271 | 1.521 |
| Quadratic term of baseline age | 0.998 | <0.001 | 0.998 | 0.999 |
| Baseline age (linear and quadratic terms) ^a^ | / | <0.001 | / | / |
| Smoking status | 1.191 | 0.013 | 1.037 | 1.367 |
| Residence | 0.757 | <0.001 | 0.710 | 0.806 |
| BMI | 0.998 | 0.013 | 0.997 | 1.000 |
| Functional status | 1.215 | <0.001 | 1.154 | 1.279 |
| Comorbidity count | 0.851 | <0.001 | 0.818 | 0.886 |
| Self-reported current exercise habits ×Soot removal (per SD) | 0.984 | 0.259 | 0.955 | 1.012 |

Note: ^a^ Baseline age was modeled with linear and quadratic terms (Wald joint test for both terms). SD: standard deviation. One SD for soot removal=5,481.5 thousand tons/year. Reference categories: no exercise habits (value = 0) for self-reported current exercise habits, male (value =1) for sex, non-smoker (value = 0) for smoking status, and rural (value =3) for residence.

**Supplementary Table 11 Sensitivity Analyses for SO_2_ Removal, Smoke Emissions and Smoke Removal**

|  | **Sensitivity analysis strategy** | **Self-reported current exercise habits HR (95%CI)** | ***P*-value for interaction ^a^** |
| --- | --- | --- | --- |
| SO_2_ removal (per SD) | Cumulative average model | 0.834(0.769-0.904)* | *P*=0.563 |
|  | LOCF model | 0.824(0.759-0.895)* | *P*=0.781 |
|  | Area‑standardized model | 0.823(0.757-0.894)* | *P*=0.379 |
|  | Reverse causation model | 0.845(0.770-0.928)* | *P*=0.501 |
| Soot emission (per SD) | Cumulative average model | 0.836(0.772-0.906)* | *P*=0.296 |
|  | LOCF model | 0.837(0.772-0.907)* | *P*=0.330 |
|  | Area‑standardized model | 0.840(0.773-0.914)* | *P*=0.744 |
|  | Reverse causation model | 0.858(0.785-0.938)* | *P*=0.226 |
| Soot removal (per SD) | Cumulative average model | 0.837(0.772-0.908)* | *P*=0.847 |
|  | LOCF model | 0.843(0.774-0.917)* | *P*=0.444 |
|  | Area‑standardized model | 0.859(0.793-0.930)* | *P*=0.721 |
|  | Reverse causation model | 0.892(0.817-0.973)* | *P*=0.115 |

Note: ^*^ *P*<0.001. ^a^ Interaction term between Self-reported current exercise habits and the respective pollution indicator. SD: standard deviation. All sensitivity analyses used the fully adjusted Model D specification. One SD for SO_2_ removal=175.1 thousand tons/year. One SD for soot emission=46.8 thousand tons/year. One SD for soot removal=5,481.5 thousand tons/year.

**Supplementary Table 12 Post‑hoc statistical power for detecting interaction effects**

| **Assumed interaction HR** | **Statistical power** |
| --- | --- |
| 1.10 | 97.73% |
| 1.15 | >99.99% |
| 1.20 | >99.99% |

Note: Power was estimated for the Wald test of the exercise with air pollution interaction term (*df* = 1) in Cox proportional hazards models.
